# Long-term results of a nationwide general ultrasound screening system for developmental disorders of the hip: the Austrian hip screening program

**DOI:** 10.1007/s11832-014-0555-6

**Published:** 2014-01-22

**Authors:** Christoph Thallinger, Renata Pospischill, Rudolf Ganger, Christof Radler, Christoph Krall, Franz Grill

**Affiliations:** 1 Orthopaedic Hospital Vienna Speising, Speisinger Strasse 109, 1130 Vienna, Austria; 2 Center for Medical Statistics, Medical University of Vienna, Spitalgasse 23, 1090 Vienna, Austria

**Keywords:** DDH, Hip sonography, Treatment rate, Open reduction rate

## Abstract

**Background:**

Diagnosis and early treatment of developmental dysplasia of the hip (DDH) continue to be issues of discussion. In 1992, a nationwide general ultrasound screening program using Graf technique was introduced to detect DDH in Austria. We investigated the effects of this program on the rates of operative and conservative interventions and the influence of the program on the number of hospital admissions for the treatment of DDH.

**Methods:**

All cases of DDH documented in Austrian hospitals from 1992 to 2008 were included in this retrospective study. The database of the Austrian Ministry of Health was used to extract documented diagnoses and treatments.

**Results:**

Since the introduction of the screening program, the number of patients who require pelvic surgery to treat DDH has decreased by 46 % and the number of open reductions is as low as 0.16 per 1,000 live births. Hospital admissions for the treatment of DDH decreased from 9.5 to 3.6 per 1,000 live births. All noted results gained statistical significance.

**Conclusion:**

Compared with routine clinically based screening programs, our results confirm low numbers of open reductions and pelvic surgeries. We, therefore, advocate a standardized nationwide general ultrasound screening program to reduce the rates of operative interventions and hospital admissions associated with the treatment of DDH.

**Level of evidence:**

Level III, diagnostic

## Introduction

Until the late 1980s, detection of developmental dysplasia of the hip (DDH) was based on voluntary clinical examinations performed by a pediatrician or an orthopaedic specialist at the time when the patient was 3–6 months old. The range of hip abduction and instability were tested with Ortolani and Barlow signs. In cases of unclear diagnosis, radiography of the pelvis was performed. General use of ultrasound for hip screening within the first days or weeks after birth began in the early 1980s. Early reports showed promising results [1, 2].

The nationwide Austrian hip screening program was introduced in 1992. It consists of clinical examination and sonographic static and dynamic imaging of the hips using the method presented by Graf [3]. With this program, two examinations are scheduled: the first sonogram shortly after birth and the second at the age of 6–8 weeks [4]. Debate still continues regarding how to screen for DDH. Some authors believe that clinical examination is sufficient [5]. Others report data on the importance of selective [6–8] or general ultrasound screening programs [9–13]. No consensus has been reached regarding how to define a pathological hip, the natural course of a dysplastic hip joint, the significance of morphological abnormalities of the hip, or which pathology should be diagnosed. It is still unclear if only unstable or dislocated hips should be diagnosed, or dysplastic hip joints should be detected as well in order to prevent osteoarthritis. Prospective randomized blinded studies to assess the natural course of DDH are not available, and no evidence of the best screening policy and method has been presented. A meta-analysis of publications in the English language literature on DDH screening conducted by the US Preventive Services Task Force [14] concluded, “Screening with clinical examination or ultrasound can identify newborns at increased risk for DDH, but because of the high rate of spontaneous resolution of neonatal hip instability and dysplasia and the lack of evidence of the effectiveness of intervention on functional outcomes, the net benefits of screening are not clear.”

Evidence for the effectiveness of a screening program could be adduced by evaluating the results of a nationwide screening program with a timeline spanning several years. We report the first nationwide database analysis and present the results of the Austrian hip screening program from 1992 to 2008. Our goal was to provide answers to the following questions: Does ultrasound screening for DDH lead to over-diagnosis and increased conservative treatment rates? Could the number of first hospital admissions be reduced? Could the number of first surgical procedures be reduced?

## Patients and methods

Data were collected from the Austrian Federal Ministry of Health, the Austrian Health Institute (known as *ÖBIG*), which is an agency that monitors and controls the Austrian Health Care system, and the Main Association of Austrian Social Security Institutions. Only data from these sources were included for patients who were treated for DDH. To search diagnosis-related codes, we used the International Classification of Diseases, Ninth Revision (ICD-9) code 754.3 up to the year 2001 and ICD, Tenth Revision (ICD-10) codes Q65.0 through Q65.8 from 2001 through 2008. These codes refer to hip dysplasia and congenital hip dislocation and are listed in Table 1.

**Table 1:** ICD-10 codes referring to hip dysplasia and congenital hip dislocation

ICD-10 code	Disease
Q65.0	Congenital dislocation of the hip; unilateral
Q65.1	Congenital dislocation of the hip; bilateral
Q65.2	Congenital dislocation of the hip; unspecified
Q65.3	Congenital subluxation of the hip; unilateral
Q65.4	Congenital subluxation of the hip; bilateral
Q65.5	Congenital subluxation of the hip; unspecified
Q65.6	Unstable hip
Q65.8	Other congenital deformities of the hip

For analysis of treatment, it was important to obtain the exact number of treated patients, not the number of cases, because only the first hospital admission or surgery was to be included in the study. To test for statistical significance of the results, Poisson regression tests were conducted to identify trends during the timeline. For data interpretation, the influence and impact of the development of the annual birthrate, immigration of children from countries without ultrasound screening of the hip, and the number of “medical tourists” who came to Austria just for hip surgery had to be evaluated.

The total number of inpatient first admissions (ICD-9 code 754.3 and ICD-10 codes Q65.0 through Q65.8) per year was calculated. Subgroups of patients from birth to age 2 years and from birth to age 4 years were formed.

Four types of surgical intervention were classified for separate evaluation: type 1, open reduction (MEL 4223); type 2, acetabuloplasty (MEL 4222); type 3, pelvic osteotomy (MEL 4206); and type 4, periacetabular osteotomy and/or triple osteotomy (MEL 4211). The number of surgeries was calculated per 1,000 births per year. Data concerning open reduction in all age groups are available from 1991 onward, and for the age group from birth to age 4 years as of 1993. For pelvic osteotomy and triple osteotomy, the oldest available data date back to 1992. Data for acetabuloplasties have been documented since 1993. Poisson regressions were calculated to detect trends in the development of rates. Adaptation of the *p* value for simultaneous hypothesis testing was accomplished by Bonferroni correction. Predictors from these Poisson regressions were included in the plots.

## Results

**Fig. 1:**
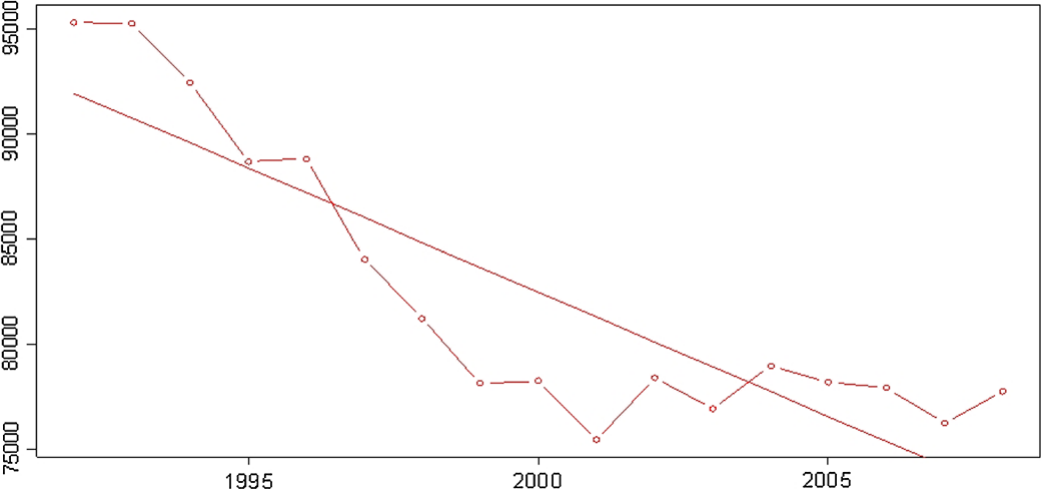
Total number of births in Austria

The birthrate in Austria has been declining over the years (Fig. 1). This has to be taken into account when measuring the clinical results.

### Does ultrasound screening for DDH lead to over-diagnosis and increased conservative treatment rates?

In 2008, the occurrence of conservative treatment, defined as use of Pavlik harness, abduction splint, and plaster cast, was 2.6 %.

### Could the number of first hospital admissions be reduced?

The number of first admissions to the hospital because of hip dysplasia and/or hip dislocation for all age groups studied declined by 62 %, from 9.5 to 3.6 per 1,000 live births during the timeline. The decrease was statistically significant, and significance remained after testing with Bonferroni correction (*p* < 0.001). The full potential of the screening program was observed 2 years after its introduction.

**Fig. 2:**
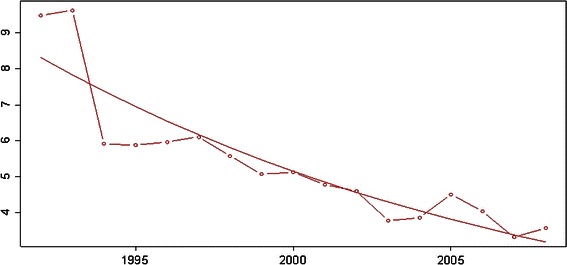
Inpatient first admissions per 1,000 births in all age groups. **a** Per region. **b** In Austria

**Fig. 3:**
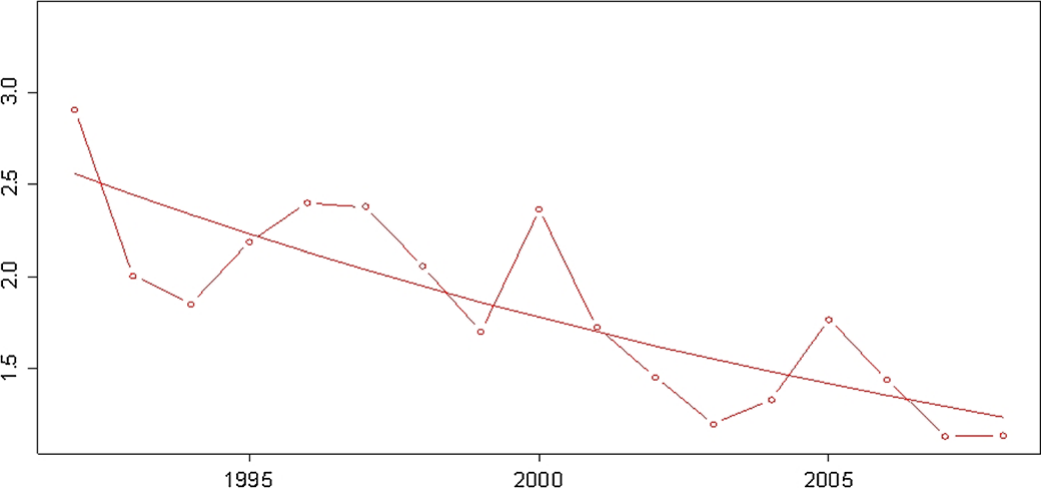
Inpatient first admissions per 1,000 births in patients from birth to age 4 years. **a** Per region. **b** In Austria

Figures 2a, b and 3a, b show the decline in numbers of first admissions to the hospital for treatment of DDH (ICD-9 code 754.3 and ICD-10 codes Q65.0 through Q65.8).

### Could the number of first surgical procedures be reduced?

Between 1992 and 2008, the number of surgical interventions (acetabuloplasty, pelvic osteotomy, triple osteotomy, periacetabular osteotomy) declined from 1.3 to 0.7 per 1,000 live births, a reduction of 46 %. It is notable that between 1993 and 1995, during the introduction phase of the use of ultrasound for screening, there was a decrease in numbers significantly stronger than during the years from 1995 to 2008 because of an overlap from prescreening years (*p* = 0.026). However, significance did not persist after multiple hypotheses testing. Nevertheless, the strong influence of ultrasound screening on number of surgeries is shown by the strong decrease in numbers. Such an alteration could not be observed for open reductions. The number of open reductions remained unchanged at 0.23 per 1,000 live births (including all age groups and all cases from unscreened pools), which is a very low level. A total of 342 open reductions were performed in the group of patients from birth to 4 years. This group includes Austrian-born patients and patients from other countries. Among these, 74 (22 %) interventions were performed in children born outside Austria in countries without ultrasonography services for screening the hip, and 44 (13 %) were performed in children born in countries with ultrasound screening (e.g., Germany, Czech Republic). The number of open reductions performed in children born in Austria was, therefore, 224. Considering only the age group younger than 4 years, the number of open reductions was as low as 0.16 per 1,000 live births. The relatively high number of children from an unscreened population born outside Austria and the relatively high percentage of children coming just for the intervention from abroad leads to a further decrease of the number of open reductions in children born in Austria to 0.12 per 1,000 live births.

## Discussion

Developmental dysplasia of the hip and its natural history is still not well understood. The term encompasses a disease spectrum ranging from a stable hip with a mildly dysplastic acetabulum to complete hip dislocation. A clinical hip screening based just on diagnosing hip instability using the Ortolani or Barlow test and asymmetry in abduction detects just the tip of the iceberg, namely only the dislocated, subluxated or unstable hips. Hip pathomorphology without instability cannot be diagnosed without the use of an imaging technique. The significance of morphological abnormalities is unknown and one may still believe that sonographically diagnosed dysplastic hips are simply immature hips that will mature independently. But there is evidence that this may not be the case. Engesaeter et al. [15] published a report on the prevalence of radiological features associated with hip dysplasia in a population of 2,081 19-year-old Norwegians. A center-edge angle of <20° was seen in 3.3 % of the cohort. Ipach et al. [16] showed that hip arthroplasty in young adults is mostly indicated because acetabular dysplasia might be the cause of the onset of osteoarthritis. This is supported by Clohisy et al. [17] who found that 48 % of 337 patients <50 years of age undergoing hip arthroplasty had had acetabular dysplasia. Engesaeter et al. [18] also reported that, unexpectedly, only 8 % of those who underwent THR due to dysplasia were reported to have had unstable hips at birth. Lee et al. [19] concluded that in 209 of 311 patients who underwent periacetabular osteotomy, acetabular dysplasia had not been diagnosed before adolescence.

This allows for drawing the conclusion that a screening program which is focused only on diagnosing unstable or dislocated hips is unable to detect acetabular dysplasia and to prevent surgical interventions like periacetabular osteotomy or hip arthroplasty at young age.

Because of this, in 2011, a selective neonatal ultrasound screening was recommended by the ESPR (European Society of Paediatric Radiology) task force group on DDH with an indication based on family history of DDH, breech presentation and positive clinical findings. It was estimated that between 12 and 16 % of all newborns will have been defined as "at risk". This recommendation was mainly based on two randomised controlled trials from Scandinavia [8, 12].

Clarke et al. [20] reported an indication for selective ultrasound screening in 18 % due to clinical signs, and in 3.6 % due to risk factors, with a treatment occurrence of 7.2/1,000 and an incidence of late presented cases of 0.34/1,000. Selective screening based on risk factors has been also proposed by Myers et al. [5]. Breech position, family history, female gender, oligohydramnios, congenital anomalies, and primiparity were considered to be risk factors. Evidence, however, is valid for positive family history only in first-degree family members [19].

There is no consensus about the value and significance of risk factors, however. Commonly known risk factors were not clinically important markers of DDH. Risk factors for DDH did not predict hip dysplasia in adolescents and adults. Only 16 % of patients undergoing periacetabular osteotomy had risk factors while ultrasound screening of a risk group did not reduce the incidence of surgery [21].

Although a selective ultrasound screening may decrease the number of surgical treatments for infant dysplasia and instability, its impact on the incidence of DDH and surgical treatment in skeletally mature patients is rather uncertain.

In conclusion, this means that there is a group of patients with acetabular dysplasia who had stable hips and no risk factors at birth but later presented with a painful hip in adolescence or early adulthood, or they were still asymptomatic but met radiographic criteria with a center-edge angle <20° [15].

Lee et al. [19] raised the hypothesis that this group represents a milder variant of infantile DDH that eluded detection at birth, or another distinct form of hip disease. Infantile dysplasia entails dislocated, subluxated, or unstable hips, which are diagnosed by neonatal physical examination. Adolescent-diagnosed and adult-diagnosed dysplasia are related to acetabular malformation that is diagnosed radiographically after symptoms develop. This idea is not new. In 1970 Wynne-Davis et al. [22] were the first to postulate that there may be two distinct types of hip dysplasia—a group with joint laxity that results in neonatal hip instability, and a group with dysplasia of late onset. A genetic disposition is discussed but could be proven in few cases only [23] .

There is no consensus how "late onset" is defined and how, and if mild/moderate dysplasia is classified on radiographs. In most studies the term is used just for missed cases of hip dislocation which had to be treated by open reduction after walking age. Only a few prospective studies with a follow-up of more than 20 years, or retrospective population studies reporting national healthcare data are published [5, 9]. The question is whether cases of acetabular dysplasia with a “potential for late presentation” could be detected by a screening program which is based on clinical testing for instability and on ultrasound for detection of dysplasia. As randomized prospective studies would not fulfil the requirements for ethical approval, only population-based studies with analysis of all available national healthcare data concerning DDH could give an answer. We are reporting the results of the Austrian neonatal hip screening program which consists of clinical tests and ultrasound since its introduction in 1992.

Our study has several limitations. A nationwide study cannot be conducted in a prospective academic high-quality setting. The study is a retrospective register-based nationwide report which reflects the reality of detection and treatment of DDH in the Austrian population.

Another limitation is the lack of reliable data concerning the time before the hip sonography screening program has been introduced. A standardized and computer registered coding system for diagnosis and treatment was not available in Austria before 1992. In the same year, the hip sonography screening program was introduced in national health care as an examination for newborns (Mutter–Kind Pass, “Mother child passport”). Therefore, statistical data of a longer timeline reaching back to the 1980s were not available to allow a comparison of the results with the previous period and to deliver even clearer results in favour of the screening program. However, for interpretation of the statistical data, 1992 was not time zero for hip sonography screening. Since 1980, after the introduction of hip sonography by Graf et al. [24], a continuously growing number of health care institutions and paediatricians or orthopaedic physicians provided sonographic examinations of newborns in Austria. Therefore, the newborn population delivered shortly before 1992 was already a voluntary screened one. Therefore, the screening results have to be compared with published data from other relevant studies.

### Conservative treatment

In central Europe a treatment occurrence of 2.6 % seems to be a reasonable number in relation to the prevalence of hip dysplasia (including dislocation and instability) [1, 3]. The MBRN (Medical Birth Registry of Norway) reported a neonatal hip instability incidence of 0.88 % between 1967 and 2004 [18]. The Norwegian data about the prevalence of hip dysplasia in a cohort of 19-year-old patients by radiographic classification report a center-edge angle <20° in 3.3 %. A prevalence of sonographically classified pathological hips (type IIc, IId, III, IV according to the Graf classification system) is reported between 1.3 and 2.4 % [25–27]. There is evidence that a certain percentage of pathological hips will grow to be normal spontaneously. However, this is a matter of fact in any prevention program and cannot be counted as overtreatment.

The concern that any abduction treatment of the dislocated hip bears the risk of avascular necrosis of the femoral head was contradicted in a Norwegian study which reported an incidence of 0 % in a collective of 2,038 newborns using abduction splints [28, 29] .

### Reduction of number of hospital admissions

The impact of a general trend to outpatient treatment of paediatric orthopaedic diseases is responsible for a certain part of the decrease of hospital admissions. The main reason, however, for the significant reduction is that neonatal ultrasound screening allows early diagnosing hip pathology, which results in shorter and less invasive kinds of treatment [13, 25, 30].

### Reduction of number of first surgical interventions—open reduction

**Fig. 4:**
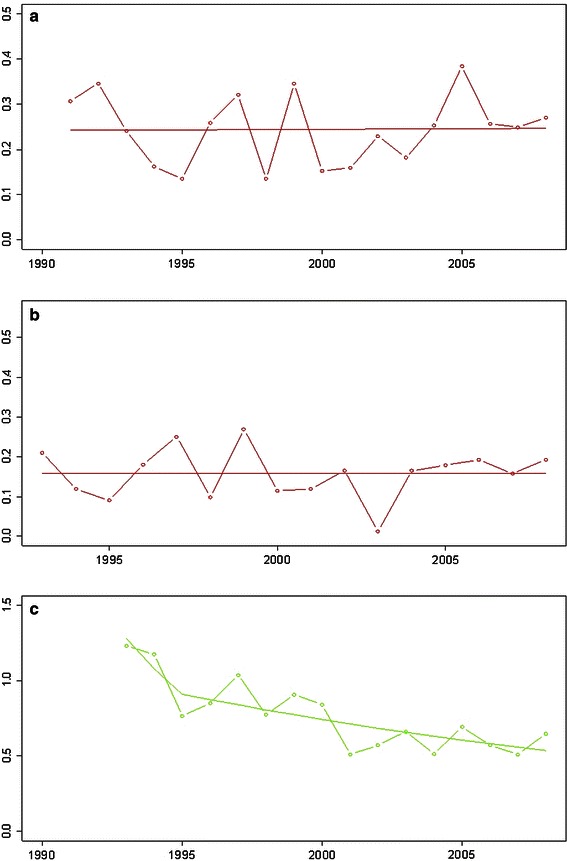
**a** First open reductions (OP) per 1,000 births, including native Austrians and immigrants. A very low number is shown for all age groups. **b** First open reductions per 1,000 births in patients from birth to age 4 years. **c** First acetabuloplasties, pelvic osteotomies, and triple osteotomies per 1,000 births

**Fig. 5:**
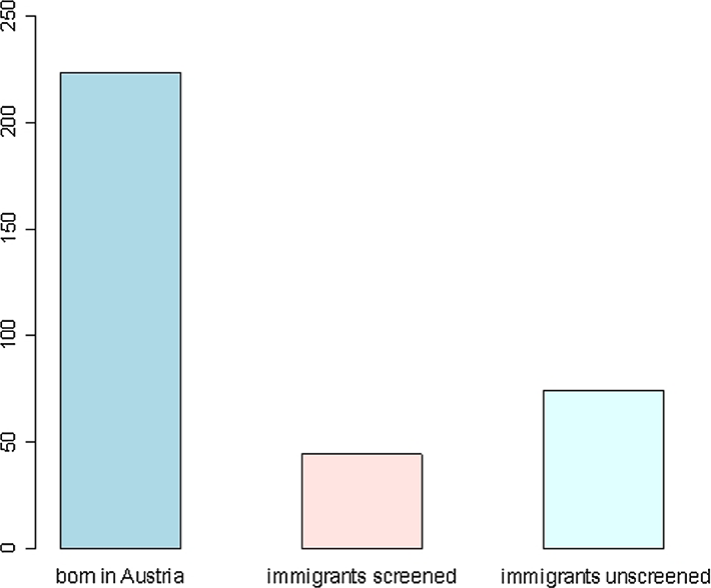
Open reductions in screened and unscreened patients

The number of open reductions could be reduced to 0.23 per 1,000 newborns including all open reductions regardless of age and whether they had undergone hip sonography screening or not. Considering only the age group younger than 4 years, the number of open reductions was as low as 0.16 per 1,000 live births. Excluding the relatively high number of children from an unscreened population born outside Austria, the number was 0.12 per 1,000 live births (Fig. 4a, b). Reported numbers of open reduction vary from 0.15 to 3.00 depending on the screening program used (Fig. 5; Table 2) [5–13, 32–35].

**Table 2:** Comparison of number of open reductions achieved with different screening programs

Screening	Open reductions per 1,000 live births	Mean
General ultrasound screening	0.23^a^ 0.26 [9] 0.15 [10] 0.33 [11] 0.3 [12] 0.41 [13]	0.28
Selective ultrasound screening	0.56 [6] 0.87 [7] 0.65 [8] 0.7 [12]	0.7
Clinical screening	0.29 [5] 1.30 [6] 1.03 [32] 0.78 [33] 3.00 [34] 1.25 [35]	1.28

^a^Current study

### Reduction of number of pelvic osteotomies and periacetabular osteotomies

Figure 4c shows a significant (*p* < 0.001) decline of pelvic osteotomies and acetabuloplasties. The decline was stronger during the 2 years after the introduction of the screening than during the following years. A progressing decrease in the number of pelvic-osteotomies of 46 % between 1992 and 2008 is promising. Further reduction is a reliable prediction. This would prove our hypothesis that the term DDH does not comprise two different pathologic entities which develop at different ages. The “adolescent-adult type of DDH” is already present at birth and could be detected by sonography. Up to now, there are no reports about patients without an underlying neuromuscular disease and with a history of a normal hip sonography at birth, but acetabular dysplasia and subsequent necessary pelvic osteotomy.

### Timing for ultrasound screening

Another controversial issue is the first hip sonography investigation during the first week after birth. Despite the fact that the screening is a scheduled examination in Austria, not every hospital provides that service.

Another fact to be taken into consideration is that the number of 1-day admission births, home births, and births in private clinics not providing orthopaedic service, is increasing. It also has to be noted that routine sonographic examinations at birth might deliver doubtful results, such as immature-looking hips that usually resolve spontaneously within the first weeks of life. The prevalence of Graf type IIa hips in neonates is approximately 20 %, which bears a potential risk for overtreatment [26]. Hip joints that are classified as type IIa look like dysplastic hip joints but are normal at that age. It is known that at 12 weeks after birth, only 11 % of former type IIa hip joints remain, being classified as type IIb from that time on [31].

Based on our findings, the ideal time for universal hip screening, including sonography, is 4–6 weeks after birth. This recommendation is also supported by von Kries et al. [13] and Grill et al. [32]. At that time, it is easy to provide a universal high-quality sonographic examination because the babies can be brought to selected screening centers with specifically trained staff. Only those who have clinically obvious pathological abnormalities and those who have a positive first-degree family history of DDH require an earlier sonographic examination, which should be manageable for the smaller patient group.

## Conclusion

The results of the Austrian hip sonography screening program show a distinct and progressing decrease of hip surgeries in adolescents and young adults. Our data support the effectiveness of this program and the hypothesis that by using ultrasound, the so-called adolescent and young adult type of DDH could be detected as well. A selective screening program may just be compromising.

Based on the results of our study, general ultrasound screening of the hip at the age of 4–6 weeks, together with a clinical examination must be recommended.

## References

[1] Tonnis D, Storch K, Ulbrich H (1990). Results of newborn screening for CDH with and without sonography and correlation of risk factors. J Pediatr Orthop.

[2] Dorn U (1990). Hip screening in newborn infants. Clinical and ultrasound results. Wien Klin Wochenschr Suppl.

[3] Graf R (2007). The use of ultrasonography in developmental dysplasia of the hip. Acta Orthop Traumatol Turc.

[4] Farr S, Grill F, Muller D (2008) When is the optimal time for hip ultrasound screening? Orthopade 37(6):532, 534–536, 538–540 10.1007/s00132-008-1236-2 18483720

[5] Myers J, Hadlow S, Lynskey T (2009). The effectiveness of a programme for neonatal hip screening over a period of 40 years: a follow-up of the New Plymouth experience. J Bone Joint Surg Br.

[6] Vane AG, Gwynne Jones DP, Dunbar JD, Theis JC (2005). The diagnosis and management of neonatal hip instability: results of a clinical and targeted ultrasound screening program. J Pediatr Orthop.

[7] Paton RW, Hossain S, Eccles K (2002). Eight-year prospective targeted ultrasound screening program for instability and at-risk hip joints in developmental dysplasia of the hip. J Pediatr Orthop.

[8] Holen KJ, Tegnander A, Bredland T, Johansen OJ, Saether OD, Eik-Nes SH, Terjesen T (2002). Universal or selective screening of the neonatal hip using ultrasound? A prospective, randomised trial of 15,529 newborn infants. J Bone Joint Surg Br.

[9] Ihme N, Altenhofen L, von Kries R, Niethard FU (2008) Hip ultrasound screening in Germany. Results and comparison with other screening procedures. Orthopade 37(6):541–546, 548–549 10.1007/s00132-008-1237-1 18491073

[10] Nimityongskul P, Hudgens RA, Anderson LD, Melhem RE, Green AE, Saleeb SF (1995). Ultrasonography in the management of developmental dysplasia of the hip (DDH). J Pediatr Orthop.

[11] Gunther KP, Stoll S, Schmitz A, Niethard FU, Altenhofen L, Melzer C, von Kries R (1998). Initial results of the evaluation study of ultrasound hip screening in Germany. Z Orthop Ihre Grenzgeb.

[12] Rosendahl K, Markestad T, Lie RT (1994). Ultrasound screening for developmental dysplasia of the hip in the neonate: the effect on treatment rate and prevalence of late cases. Pediatrics.

[13] von Kries R, Ihme N, Altenhofen L, Niethard FU, Krauspe R, Ruckinger S (2012). General ultrasound screening reduces the rate of first operative procedures for developmental dysplasia of the hip: a case-control study. J Pediatr.

[14] Shipman SA, Helfand M, Moyer VA, Yawn BP (2006). Screening for developmental dysplasia of the hip: a systematic literature review for the US preventive services task force. Pediatrics.

[15] Engesaeter IO, Laborie LB, Lehmann TG, Fevang JM, Lie SA, Engesaeter LB, Rosendahl K (2013). Prevalence of radiographic findings associated with hip dysplasia in a population-based cohort of 2081 19-year-old Norwegians. Bone Joint J.

[16] Ipach I, Mittag F, Syha R, Kunze B, Wolf P, Kluba T (2012). Indications for total hip arthroplasty in young adults—idiopathic osteoarthritis seems to be overestimated. Rofo.

[17] Clohisy JC, Dobson MA, Robison JF, Warth LC, Zheng J, Liu SS, Yehyawi TM, Callaghan JJ (2011). Radiographic structural abnormalities associated with premature, natural hip-joint failure. J Bone Joint Surg Am.

[18] Engesaeter IO, Lie SA, Lehmann TG, Furnes O, Vollset SE, Engesaeter LB (2008). Neonatal hip instability and risk of total hip replacement in young adulthood: follow-up of 2,218,596 newborns from the Medical Birth Registry of Norway in the Norwegian Arthroplasty Register. Acta Orthop.

[19] Lee CB, Mata-Fink A, Millis MB, Kim YJ (2013). Demographic differences in adolescent-diagnosed and adult-diagnosed acetabular dysplasia compared with infantile developmental dysplasia of the hip. J Pediatr Orthop.

[20] Clarke NM, Reading IC, Corbin C, Taylor CC, Bochmann T (2012). Twenty years experience of selective secondary ultrasound screening for congenital dislocation of the hip. Arch Dis Child.

[21] Eastwood DM (2003). Neonatal hip screening. Lancet.

[22] Wynne-Davies R (1970). Acetabular dysplasia and familial joint laxity: two etiological factors in congenital dislocation of the hip. A review of 589 patients and their families. J Bone Joint Surg Br.

[23] Okano K, Takaki M, Okazaki N, Shindo H (2008). Bilateral incidence and severity of acetabular dysplasia of the hip. J Orthop Sci.

[24] Graf R, Tschauner C (1994). Sonography of the infant hip. Sources of error, progress and current clinical relevance. Radiologe.

[25] Wirth T, Stratmann L, Hinrichs F (2004). Evolution of late presenting developmental dysplasia of the hip and associated surgical procedures after 14 years of neonatal ultrasound screening. J Bone Joint Surg Br.

[26] Schilt M (2001). Optimal age for hip sonography screening. Ultraschall Med.

[27] Falliner A, Schwinzer D, Hahne HJ, Hedderich J, Hassenpflug J (2006). Comparing ultrasound measurements of neonatal hips using the methods of Graf and Terjesen. J Bone Joint Surg Br.

[28] Grill F, Bensahel H, Canadell J, Dungl P, Matasovic T, Vizkelety T (1988). The Pavlik harness in the treatment of congenital dislocating hip: report on a multicenter study of the European Paediatric Orthopaedic Society. J Pediatr Orthop.

[29] Laborie LB, Engesaeter IO, Lehmann TG, Eastwood DM, Engesaeter LB, Rosendahl K (2013). Screening strategies for hip dysplasia: long-term outcome of a randomized controlled trial. Pediatrics.

[30] Tschauner C, Klapsch W, Baumgartner A, Graf R (1994). Maturation curve of the ultrasonographic alpha angle according to Graf’s untreated hip joint in the first year of life. Z Orthop Ihre Grenzgeb.

[31] Schule B, Wissel H, Neumann W, Merk H (1999). Follow-up control of ultrasonographic neonatal screening of the hip. Ultraschall Med.

[32] Grill F, Muller D (1997). Results of hip ultrasonographic screening in Austria. Orthopade.

[33] Chang CH, Chiang YT, Lee ZL, Kuo KN (2007). Incidence of surgery in developmental dysplasia of the hip in Taiwan. J Formos Med Assoc.

[34] Maxwell SL, Ruiz AL, Lappin KJ, Cosgrove AP (2002). Clinical screening for developmental dysplasia of the hip in Northern Ireland. BMJ.

[35] Godward S, Dezateux C (1998). Surgery for congenital dislocation of the hip in the UK as a measure of outcome of screening. MRC working party on congenital dislocation of the hip. Medical Research Council. Lancet.

